# Study on the Direct and Indirect Photolysis of Antibacterial Florfenicol in Water Using DFT/TDDFT Method and Comparison of Its Reactivity with Hydroxyl Radical under the Effect of Metal Ions

**DOI:** 10.3390/toxics12020127

**Published:** 2024-02-03

**Authors:** Yue Kang, Ying Lu, Se Wang

**Affiliations:** Collaborative Innovation Center of Atmospheric Environment and Equipment Technology, Jiangsu Key Laboratory of Atmospheric Environment Monitoring and Pollution Control, School of Environmental Science and Engineering, Nanjing University of Information Science and Technology, Nanjing 210044, China; 202083280094@nuist.edu.cn (Y.K.); 20211248120@nuist.edu.cn (Y.L.)

**Keywords:** antibacterials, florfenicol, photolysis, metal ion influence, DFT

## Abstract

Florfenicol (FLO) is a widely used antibacterial drug, which is often detected in the environment. In this paper, the photolysis mechanism of FLO in water was investigated using density functional theory (DFT) and time-dependent density functional theory (TDDFT). The focus of the study is to elucidate the direct photolysis mechanism of FLO in the water environment and the indirect photolysis of free radicals (·OH, ·NO_3_, and ·SO_4_^−^) as active species. The effect of metal ions Ca^2+^/Mg^2+^/Zn^2+^ on the indirect photolysis was also investigated. The results show that the direct photolysis of FLO involves C–C/C–N/C–S bond cleavage, the C5–S7 bond cleavage is most likely to occur, and the C17–C18 cleavage reaction is not easy to occur during the direct photodegradation of FLO. The indirect photolysis of FLO is more likely to occur in the environment than direct photolysis. The main indirect photolysis involves OH-addition, NO_3_-addition, and SO_4_-addition on benzene ring. The order of difficulty in the indirect photolysis with ·OH is C2 > C3 > C4 > C5 > C6 > C1, Ca^2+^ can promote the indirect photolysis with ·OH, and Mg^2+^/Zn^2+^ has a dual effect on the indirect photolysis with ·OH. In other words, Mg^2+^ and Zn^2+^ can inhibit or promote the indirect photolysis with ·OH. These studies provide important information for theoretical research on the environmental behavior and degradation mechanism of drug molecules.

## 1. Introduction

Florfenicol (FLO) is a broad-spectrum chloramphenicol antibiotic widely used in the prevention and treatment of bacterial diseases, which can inhibit or eliminate the growth of microorganisms [1,2,3,4,5]. During aquaculture, FLO, as a pharmaceutical feed, is usually leached directly or released into the aquatic environment with feces [6,7]. The average and maximum concentration of FLO in the water around the farm is 0.42 and 2.84 μg/L, and the maximum concentration in the Huangpu River is 46.6 ng L^−1^ [8,9]. Its residues are likely to lead to the development and spread of drug-resistant bacteria/resistance genes, which in turn limits the treatment options for human infections [10,11].

Since FLO exists in water for a long time and may pose a threat to the aquatic environment and food safety, there is an urgent need to investigate its chemical behavior in water to evaluate its risks [12,13]. The degradation of FLO in water mainly includes hydrolysis, biodegradation, and photolysis [14,15,16]. Photolysis can be divided into direct photolysis and indirect photolysis. Direct photolysis means that substances directly absorb light energy for decomposition reactions. During the indirect photolysis process, FLO can react with ·OH, O_3_, ^1^O_2_, and other reactive oxygen species in the water environment [17]. Direct and indirect photolysis play an indispensable role in the environmental behavior of drugs [14,18]. 

In recent years, some studies have focused on the degradation behavior of FLO under UV/H_2_O_2_, UV/Fe (II), and UV/Na_2_S_2_O_3_ conditions [18,19]. Xu et al. [20] studied the degradation behavior of FLO and its metabolites under different soil and natural conditions based on a simple method. In the past, photocatalysis has been shown to be an efficient treatment for the removal of aquaculture antibiotics, and it is necessary to consider changes in toxicity due to FLO decomposition [21,22]. Liu et al. [18] concluded that the toxicity of final products of FLO by UV irradiation was lower than FLO. UV irradiation-activated persulfate process can effectively control the toxicity of FLO degradation [19]. Meanwhile, photolysis is dramatically affected by environmental factors such as the type of light, pH, or antibiotic initial concentration [21,23,24]. Jiang et al. [25] considered that the superior stability and low concentration of C–F bonds in FLO hindered its degradation, and the degradation products may be as active/toxic as FLO. GE et al. [24] studied the effects of Cl^−^, humic acid (HA), and other water components on the photodegradation of antibiotics; under UV–visible irradiation, Cl^−^ can promote the formation of ^1^O_2_ and accelerate the photodegradation of phenols, while under simulated solar irradiation, its photodegradation is not related to Cl^−^; HA inhibits photolysis under UV–visible irradiation, but it can undergo photosensitive degradation under simulated solar irradiation. Laboratory experiments are time-consuming, laborious, and costly, and it is difficult to decipher all the pathways of organic pollutant degradation in a short time. More importantly, theoretical calculations can find the intermediate products of the reaction process, which is difficult to achieve with laboratory experiments. As a result, there is a lack of computational research on the water environment behavior of FLO, especially the photodegradation behavior in water environment, which has guiding significance and verification function for the laboratory study.

By the means of quantum chemical calculation, this study intends to use density functional theory (DFT) and time density functional theory (TDDFT) to explore the photochemical transformation process of FLO in water environment [26,27,28,29,30]. The focus of the study is to elucidate the direct photolysis mechanism of FLO in water and the indirect photolysis of free radicals (·OH, ·NO_3_, ·SO_4_^−^) as active species. In addition, the effects of different metal ions (Ca^2+^/Mg^2+^/Zn^2+^) on the indirect photodegradation of FLO and ·OH were also studied. The computational methods employed in this research provide valuable insights into the photodegradation pathways and reactive intermediates involved in the photolysis of FLO in aqueous environments. Additionally, these findings contribute to a better understanding of the environmental fate and the potential remediation strategies of FLO contaminants.

## 2. Computational Methods

The calculations in this study were performed using the Gaussian 16 quantum chemistry software package [31,32]. The DFT/TDDFT has been extensively tested and validated against experimental data and has been shown to provide reliable predictions for the photolysis behavior of a variety of organic compounds [33,34,35,36]. Based on the B3LYP/6-311+G (d,p) level, DFT and TDDFT were used to optimize the stable structures of FLO in the ground state (S_0_) and the lowest excited triplet state (T_1_), respectively [37,38,39]. The intrinsic reaction coordinate (IRC) was used to analyze and verify the specified reactants (R) and products (P) for each transition state (TS) linkage [40]. In all calculations, the solvent effect in water is taken into account, and the integral equation in the form of the polarized continuum model (IEFPCM) is used [39]. At the B3LYP/6-311+G(d,p) level, TDDFT theory is used to calculate the electronic absorption spectra of monomer FLO and metal ion complexes FLO–Ca^2+^/FLO–Mg^2+^/FLO–Zn^2+^ in water [41,42]. The photodegradation mechanism of FLO in aqueous solution was analyzed by calculating the atomic charge and electron spin density based on natural bond orbital (NBO) at the B3LYP/6-311+G (d,p) level of theory. It mainly includes direct photodegradation (C–C bond cleavage, C–N bond cleavage, C–S bond cleavage), indirect photodegradation with free radicals (·OH, ·NO_3_, ·SO_4_^−^) in water, and indirect photodegradation with ·OH under the effect of metal Ca^2+^/Mg^2+^/Zn^2+^.

The structures of Rs (reactants), TSs (transition states), and Ps (products) during the direct and indirect photolysis were optimized at the B3LYP/6-311+G(d,p) level. As T_1_ state has been found to be a precursor of photochemical reactions that exists for a long time, the calculation of photodegradation processes in this paper is carried out in T_1_ state [16]. Through the calculation and analysis of TS vibration, it is ensured that there is only one imaginary frequency. All the calculated activation energy (*E*_a_) and enthalpy changes (Δ*H*) of the reaction are modified by zero–point energy.

## 3. Results and Discussion

### 3.1. Optimized Geometry of FLO

**Figure 1:**
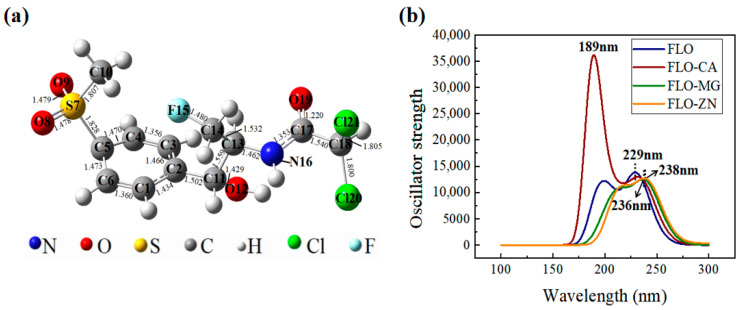
(**a**) Optimized geometries of FLO along with selected bond lengths (Å) and (**b**) calculated electronic absorption spectra of monomer FLO and complexes FLO–Ca^2+^/FLO–Mg^2+^/FLO–Zn^2+^ with maximum absorption wavelength (nm).

Figure 1a shows the optimized geometry of FLO. The calculation results indicate that although the benzene ring is symmetrical, the bond lengths are not the same. For example, the C2–C3 bond (1.466 Å) is longer than the C2–C1 bond (1.434 Å), and the C5–C4/C4–C3 bond (1.470 Å / 1.356 Å) is shorter than the C5–C6/C6–C1 bond (1.473 Å / 1.360 Å). Figure 1b shows the electronic absorption spectra of FLO. The calculated maximum absorption wavelength of FLO is 229 nm. Experimental results show that the maximum absorption wavelength of FLO is 224 nm [11]. This indicates that the theoretical calculation results are consistent with the experimental results. Due to absorption by the atmosphere, the shortest wavelength of solar radiation reaching the Earth’s surface is 290 nm [43]. In natural water, FLO can partially undergo direct photodegradation to a certain extent.

### 3.2. Direct Photolysis of FLO in Water

The direct photodegradation pathway of FLO in water with four reaction sites is shown in Figure S1. Figure S2 shows the optimized geometries of Rs, TSs, and Ps for the four reaction sites. The distance between the two sites of the broken bonds in the TSs for the four reaction sites is 1.799–2.100 Å. The Δ*H* values show that the cleavage of C11–C13/C5–S7 bond (Path 1/Path 4) is exothermic, and the cleavage of C13–N16/C17–C18 bond (Path 2/Path 3) is endothermic. The C11–C13 bond cleavage (Path 1) released the most heat (Δ*H* = −36.2 kcal/mol), indicating that the products P1a and P1(b) were the most stable.

**Figure 2:**
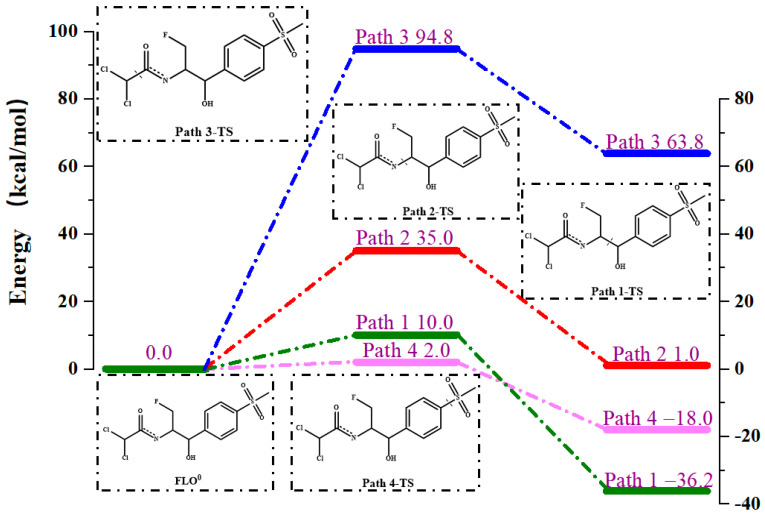
Reaction energy barrier diagram of the direct photolysis of FLO.

Pathways 1, 2, 3, and 4 involve C11–C13, C13–N16, C17–C18, and C5–S7 bond cleavage (Path 1, Path 2, Path 3, and Path 4). The energy barrier diagram of the direct photolysis of FLO is shown in Figure 2. The *E*_a_ values of C11–C13/C13–N16/C5–S7 bond cleavage (Path 1/Path 2/Path 4) of FLO in T_1_ state are 10.0 kcal/mol, 35.0 kcal/mol, and 2.0 kcal/mol, respectively, which were obviously lower than the cleavage of C17–C18 bond (*E*_a_ = 94.8 kcal/mol) (Path 3). The relatively high *E*_a_ value (94.8 kcal/mol) indicates that the C17–C18 cleavage reaction (Path 3) does not easily occur during the direct photodegradation of FLO. The *E*_a_ value (2.0 kcal/mol) for the C5–S7 bond cleavage (Path 4) is the lowest among all the four paths, indicating that C5–S7 bond cleavage (Path 4) is most likely to occur in direct photodegradation, and this reaction produces P4a and P4b products.

### 3.3. Indirect Photolysis of FLO in Water

#### 3.3.1. Indirect Photolysis Mechanism of FLO and ·OH

**Figure 3:**
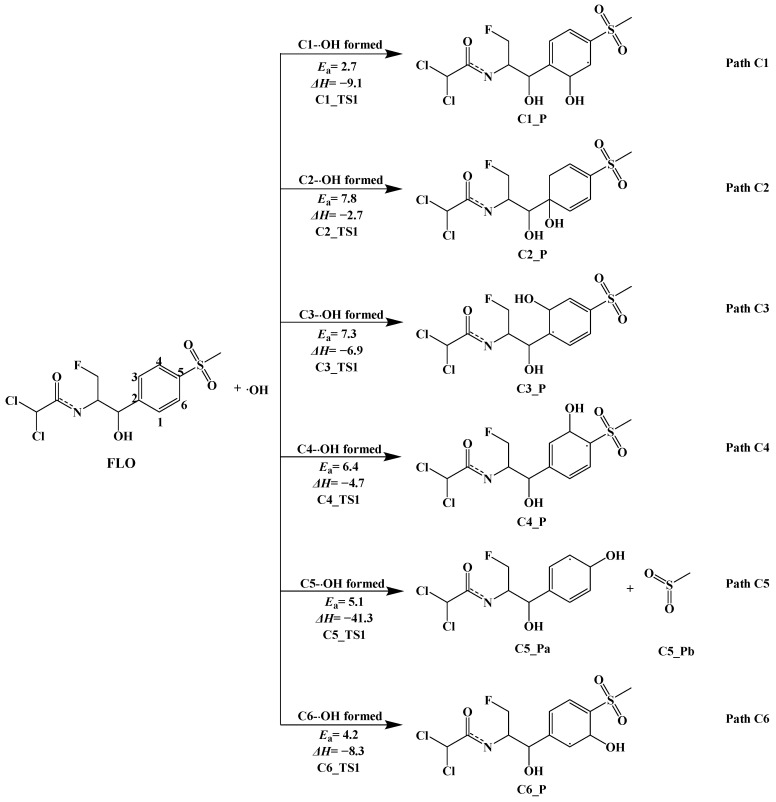
Indirect photodegradation pathways of FLO with ·OH, along with computed activation energies (*E*_a_, kcal/mol) and enthalpy change (Δ*H*, kcal/mol).

Six reaction pathways of FLO with ·OH in water were predicted, and the reaction type of these pathways was ·OH addition reaction (Figure 3). By calculation, the reactions at the C1, C2, C3, C4, C5, and C6 sites on the benzene ring were predicted to be likely to occur. Figure S3 shows the optimized geometries of Rs, TSs, and Ps at six reaction sites. The distance between ·OH and the six reaction sites in the TSs of FLO is 2.011–3.006 Å. Figure 3 shows that all the reaction pathways (Path C1, Path C2, Path C3, Path C4, Path C5, and Path C6) are exothermic, and the Δ*H* values range from −2.7 kcal/mol to −41.3 kcal/mol. The addition reaction (Path C5) at the C5 site is the most exothermic (Δ*H* = −41.3 kcal/mol), indicating that the generated products C5_Pa and C5_Pb are the most stable.

OH is predicted to attack six C sites on the benzene ring (Path C1, Path C2, Path C3, Path C4, Path C5, and Path C6) to generate C1_P, C2_P, C3_P, C4_P, C5_P, and C6_P. Zhang et al. [17] also detected that phenolic products were formed after the electrophilic attack of ·OH at the C site of benzene ring in FLO photodegradation experiments. The reaction of ·OH attacking at the C2 site (Path C2) releases the least heat (Δ*H =* −2.7 kcal/mol). *E*_a_ values range from 2.7 kcal/mol to 7.8 kcal/mol, and lower *E*_a_ values indicated that ·OH promotes the photolysis of FLO, which is consistent with the results of Li et al. [44]. The *E*_a_ value for ·OH (Path C1) attacking at the C1 reaction site is the lowest (2.7 kcal/mol), indicating that this reaction is the easiest to occur and most conducive to the formation of C1_P. In addition, since the *E*_a_ value (7.8 kcal/mol) for ·OH attacking at C2 (Path C2) is the maximum value of all the reactions of ·OH attacking at FLO, Path C2 is not conducive to occur. A similar reaction was also shown at the C3 site. The *E*_a_ value for ·OH attacking at the C3 site (Path C6) is 7.3 kcal/mol. The data with little difference indicate that C2_P and C3_P are likely to be generated simultaneously 

#### 3.3.2. Indirect Photolysis Mechanism of FLO and ·NO_3_

**Figure 4:**
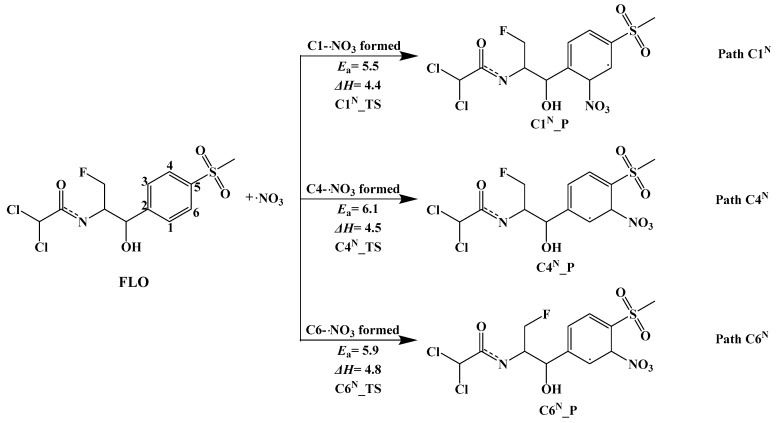
Indirect photodegradation pathways of FLO with ·NO_3_, along with computed activation energies (*E*_a_, kcal/mol) and enthalpy change (Δ*H*, kcal/mol).

Three possible pathways (Path C1^N^, Path C4^N^, and Path C6^N^) of the indirect photodegradation of FLO by ·NO_3_ in water are shown in Figure 4, and the reaction type of these pathways is an addition reaction. There are three possible reaction sites on the benzene ring: C1, C4, and C6. Figure S4 shows the geometric optimization of Rs, TSs, and Ps for the three reaction sites. The distance between ·NO_3_ and the three reaction sites in the TSs is 1.711–1.866 Å. Figure 4 shows that the three reaction pathways (Path C1^N^, Path C4^N^_,_ and Path C6^N^) are endothermic, with Δ*H* values ranging from 4.4 kcal/mol to 4.8 kcal/mol.

The relatively low *E*_a_ value (5.5 kcal/mol) indicates that ·OH is most likely to react at the C1 site (Path C1^N^) and is most conducive to the formation of C1^N^_Pa. Similar reactions were also shown at the C4 and C6 sites. The *E*_a_ values for adding ·OH at the C4 site (Path C4^N^) and C6 site (Path C6^N^) are 6.1 kcal/mol and 5.9 kcal/mol, respectively. The data with little difference show that sites C1, C4, and C6 are beneficial to react with ·OH.

#### 3.3.3. Indirect Photolysis Mechanism of FLO and ·SO_4_^−^

**Figure 5:**
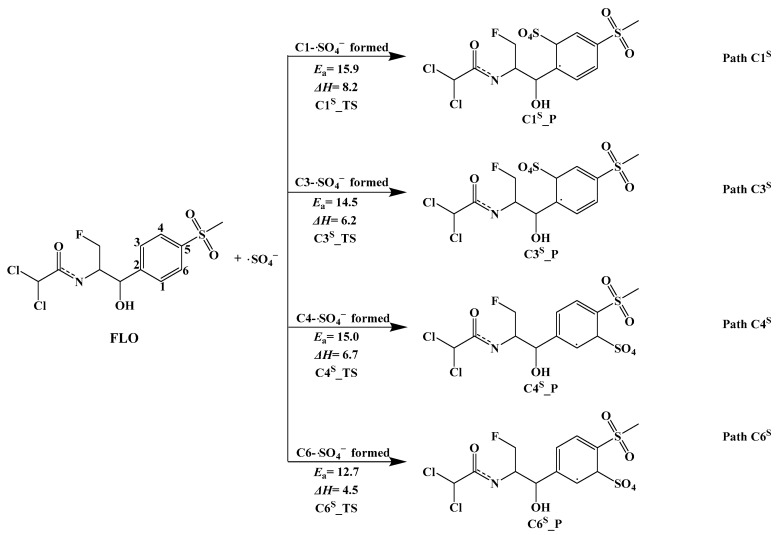
Indirect photodegradation pathways of FLO with ·SO_4_^−^, along with computed activation energies (*E_a_*, kcal/mol) and enthalpy change (Δ*H*, kcal/mol).

Four possible pathways (Path C1^S^, Path C3^S^, Path C4^S^, and Path C6^S^) of the indirect photodegradation of FLO with ·SO_4_^−^ in water are shown in Figure 5, and the reaction type is an addition reaction. Figure S5 shows the optimized geometries of Rs, TSs, and Ps for the four reaction sites. The distance between ·SO_4_^−^ and the four reaction sites in the TSs is 1.910–1.964 Å. All the pathways (Path C1^S^, Path C3^S^, Path C4^S^, and Path C6^S^) are predicted to be endothermic, with Δ*H* ranging from 4.5 kcal/mol to 8.2 kcal/mol (Figure 5).

The lowest *E*_a_ value (12.7 kcal/mol) for Path C6^S^ among all the four pathways indicates that ·OH is most likely to react at the C6 site and is most conducive to the formation of C6^S^_Pa. The *E*_a_ value for adding ·SO_4_^−^ to the C1 site was 15.9 kcal/mol (Path C1^S^), which is the maximum value of all the four pathways. This indicates that the reaction of ·SO_4_^−^ attacking at the C1 site (Path C1^S^) is not conducive to proceed.

#### 3.3.4. Optimized Geometries of Complexes FLO–Ca^2+^/FLO–Mg^2+^/FLO–Zn^2+^

Figure S6 shows the optimized geometries of complexes FLO–Ca^2+^/FLO–Mg^2+^/FLO–Zn^2+^. The calculation results show that the bond lengths of the complexes are different from those of the monomer FLO. For example, the bond lengths of S7–O9/ S7–C5/C2–C11 in complex FLO–Mg^2+^ are longer than those of FLO, conversely, the bond lengths of C11–C13 in complex FLO–Mg^2+^ are shorter than those of FLO (Figure 1a and Figure S9).

Figure 1b shows the electronic absorption spectra of complexes FLO–Ca^2+^/FLO–Mg^2+^/FLO–Zn^2+^ in water at the TDDFT/B3LYP/6-311+G(d,p) level of theory. The bond length of Ca–O8 in the complex FLO–Ca^2+^ is the longest (2.329 Å) and the maximum absorption wavelength is the smallest (189 nm) among all the three metal ions complexes. The bond length of Zn–O8 in the complex FLO–Zn^2+^ is the shortest (1.926 Å), and the maximum absorption wavelength is the largest (238 nm).

Compared with the monomer, there is a blue shift in the maximum electron absorbance peak position of complex FLO–Ca^2+^, while there is a red shift in those of the complexes FLO–Mg^2+^/FLO–Zn^2+^. Therefore, the metal ions Ca^2+^/Mg^2+^/Zn^2+^ have an effect on the structure and UV absorption spectrum of FLO and may also have an effect on the photodegradation of FLO.

#### 3.3.5. Indirect Photolysis of Complexes FLO–Ca^2+^/FLO–Mg^2+^/FLO–Zn^2+^ with ·OH

##### Mechanism of Indirect Photolysis of Complex FLO–Ca^2+^ with ·OH in Water

Figure S7 shows the optimized geometries of complex FLO–Ca^2+^. Results show that the complex FLO–Ca^2+^ has four possible geometries. By calculating the minimum value of the potential energy surface, the most stable geometric structure (FLO–Ca^2+^–OPT3) with the lowest single-point energy is obtained among the four structures (Figure S8). All of the following calculations are based on the FLO–Ca^2+^–OPT3 structure.

**Figure 6:**
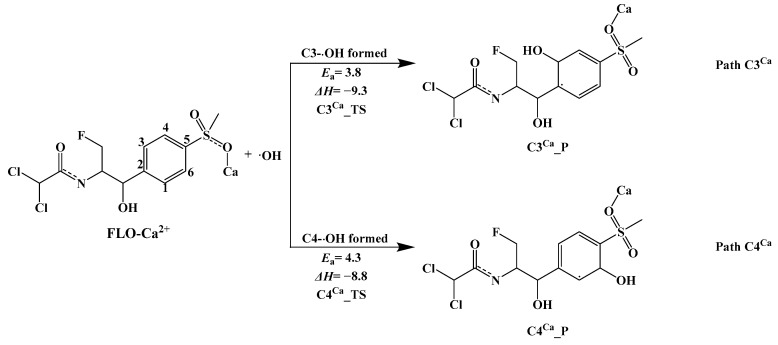
Indirect photodegradation pathways of complex FLO–Ca^2+^ with ·OH, along with computed activation energies (*E*_a_, kcal/mol) and enthalpy change (Δ*H*, kcal/mol).

Two possible pathways (Path C3^Ca^ and Path C4^Ca^) of the indirect photolysis of complex FLO–Ca^2+^ with ·OH are shown in Figure 6, and the reaction type is an addition reaction. There are two possible reaction sites on the benzene ring: C3 and C4. Figure S7 shows the optimized geometries of Rs, TSs, and Ps for the two reaction sites. The distance between ·OH and the two reaction sites in the TSs is 2.028 and 2.033 Å, and the difference is very small. Both the two pathways (Path C3^Ca^ and Path C4^Ca^) shown in Figure S7 are exothermic, with Δ*H* values ranging from −8.8 to −9.3 kcal/mol.

The lower *E*_a_ value (3.8 kcal/mol) indicates that ·OH is more likely to react at the C3 site (Path C3^Ca^) than that at the C4 site. The *E*_a_ value for adding ·OH to the C4 site (Path C4^Ca^) is 4.3 kcal/mol. For the complex FLO–Ca^2+^, the calculated *E*_a_ values for the two pathways by ·OH (Path C3^Ca^ and Path C4^Ca^) are lower than that for the indirect photolysis of monomer FLO. This indicates that Ca^2+^ can promote the ·OH addition reaction at C3 and C4 sites. In addition, as shown in Figure 3, ·OH is more prone to attack the C4 site than the C3 site for the indirect photolysis of monomer FLO. However, in the presence of Ca^2+^, the *E*_a_ value for adding ·OH to the C3 site (Path C3^Ca^, *E*_a_ *=* 3.8 kcal/mol) is lower than that for the C4 site (Path C4^Ca^, *E*_a_ *=* 4.3 kcal/mol). This indicates that Ca^2+^ can change the main indirect photolysis pathway of FLO.

##### Indirect Photodegradation Mechanism of Complex FLO–Mg^2+^ and ·OH in Water

The optimized geometries of complex FLO–Mg^2+^ are shown in Figure S9. The results show that complex FLO–Mg^2+^ has five possible geometries. The most stable geometric structure (FLO–Mg^2+^–OPT4 structure) with the lowest single-point energy is obtained among the five structures (Figure S9). All calculations are based on the FLO–Mg^2+^–OPT4 structure.

**Figure 7:**
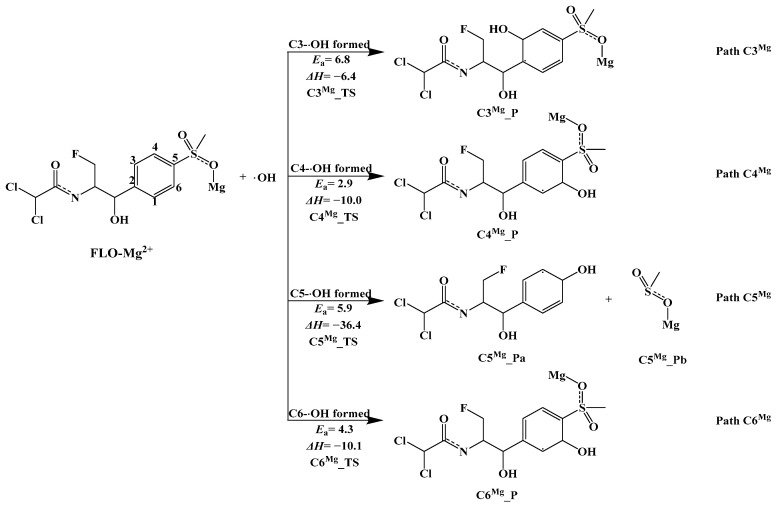
Indirect photodegradation pathways of complex FLO–Mg^2+^ with ·OH, along with computed activation energies (*E*_a_, kcal/mol) and enthalpy change (Δ*H*, kcal/mol).

Four possible pathways (Path C3^Mg^, Path C4^Mg^, Path C5^Mg^, and Path C6^Mg^) of the indirect photodegradation of complex FLO–Mg^2+^ with ·OH are shown in Figure 7, and the reaction type of these pathways is an addition reaction. Four reaction sites (C3, C4, C5, and C6) on the benzene ring were predicted to undergo reactions. Figure S10 shows the optimized geometries of Rs, TSs, and Ps for these four reaction sites. The distance between ·OH and the four reaction sites in the TSs is 1.998–2.049 Å. Figure 7 shows that the Δ*H* values for all the pathways (Path C3^Mg^, Path C4^Mg^, Path C5^Mg^, and Path C6^Mg^) range from −6.4 kcal/mol to −36.4 kcal/mol, and the four pathways are exothermic. The Δ*H* value (−36.4 kcal/mol) for ·OH attacking at the C5 site is the lowest among the four pathways, indicating that the products C5^Mg^_Pa and C5^Mg^_Pb are the most stable.

The *E*_a_ value (2.9 kcal/mol) for Path C4^Mg^ of all the four pathways is the lowest and indicates that ·OH is most likely to react at the C4 site. A similar reaction was also obtained at the C6 site. The *E*_a_ value for adding ·OH at the C6 site is 4.3 kcal/mol. In addition, the *E*_a_ value (6.8 kcal/mol) for ·OH attacking at the C3 site is the highest of all the four pathways, so Path C3^Mg^ is the most difficult for undergoing photolysis reactions with ·OH. For C3 and C4 sites, the calculated *E*_a_ values of the indirect photolysis of the complex FLO–Mg^2+^ with ·OH (Path C3^Mg^ and Path C4^Mg^) are lower than those of monomer FLO with ·OH(Path C3 and Path C4). In addition, *E*_a_ values for the Path C5^Mg^ and Path C6^Mg^ of complex FLO–Mg^2+^ are higher than those of the reaction of monomer FLO with ·OH at C5 and C6 sites(Path C5 and Path C6). This indicates that Mg^2+^ has a dual role in the photolysis of FLO. Mg^2+^ can promote ·OH attacking at the C3 and C4 sites (Path C3^Mg^ and Path C4^Mg^) and inhibit ·OH attacking at the C5 and C6 sites (Path C5^Mg^ and Path C6^Mg^). In addition, Mg^2+^ can also change the main photolysis pathway of FLO with ·OH. In the presence of Mg^2+^, the order of difficulty in the indirect photolysis with ·OH is C3 > C5 > C6 > C4, and for monomer FLO, the order is C3 > C4 > C5 > C6.

##### Mechanism of Indirect Photodegradation of Complex FLO–Zn^2+^ with ·OH in Water

The optimized geometries of complex FLO–Zn^2+^ are shown in Figure S11. The results show that the complex FLO–Zn^2+^ has four possible geometries, and the most stable geometric structure with the lowest single-point energy is FLO–Zn^2+^–OPT1 (Figure S11). Thus, all calculations are based on the FLO–Zn^2+^–OPT1 structure.

**Figure 8:**
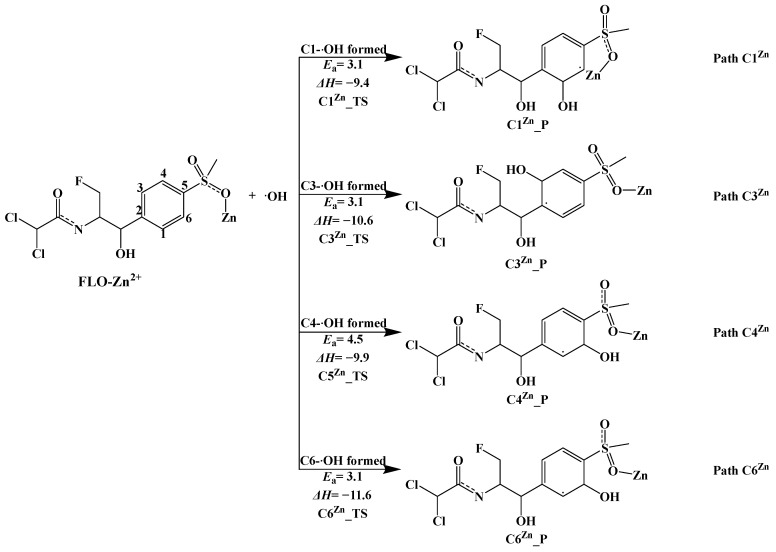
Indirect photodegradation pathways of complex FLO–Zn^2+^ with ·OH, along with computed activation energies (*E*_a_, kcal/mol) and enthalpy change (Δ*H*, kcal/mol).

Four possible pathways (Path C1^Zn^, Path C3^Zn^, Path C4^Zn^, and Path C6^Zn^) of the indirect photodegradation of the complex FLO–Zn^2+^ with ·OH are shown in Figure 8, and the reaction type is an addition reaction. Figure S12 shows the optimized geometries of Rs, TSs, and Ps for the four reaction sites (C1, C3, C4, and C6). The distance between ·OH and the four reaction sites is 2.013–2.036 Å. Figure 8 shows that Δ*H* values for all the four pathways (Path C1^Zn^, Path C3^Zn^, Path C4^Zn^, and Path C6^Zn^) range from −9.4 kcal/mol to −11.6 kcal/mol, and these pathways are exothermic. The Δ*H* value (−11.6 kcal/mol) for ·OH attacking at the C6 site is the lowest among all the four pathways, indicating that product C6^Zn^_P is the most stable.

The *E*_a_ values for ·OH attacking at the C1, C3, and C6 sites are all 3.1 kcal/mol, which are lower than that for the C4 cite (4.5 kcal/mol). This indicates that ·OH is more likely to react at the C1, C3, and C6 sites (Path C1^Zn^, Path C3^Zn^_,_ and Path C6^Zn^). In addition, the calculated *E*_a_ values for Path C3^Zn^, Path C4^Zn^, and Path C6^Zn^ of the complex FLO–Zn^2+^ are lower than those for the indirect photolysis of monomer FLO with ·OH, and the *E*_a_ value for Path C1^Zn^ of complex FLO–Zn^2+^ is higher than that for the indirect photolysis of monomer FLO with ·OH. This indicates that Zn^2+^ has a dual role in the indirect photolysis of FLO with ·OH, i.e., Zn^2+^ can promote ·OH attacking at the C3, C4, and C6 sites and inhibit ·OH attacking at the C1 site. In addition, Zn^2+^ can also change the main photodegradation pathway of FLO with ·OH, i.e., in the presence of Zn^2+^, the order of difficulty of ·OH attacking at the different reaction sites in the indirect photodegradation of FLO with ·OH is C4 > C6 = C1 = C3, which is different from that of monomer FLO (C3 > C4 > C6 > C1).

## 4. Conclusions

This study provides a basis for further understanding the photodegradation mechanism of FLO in water. These mechanisms involve direct photodegradation, indirect photodegradation with free radicals (·OH, ·NO_3_, and ·SO_4_^−^), and the effect of metal ions (Ca^2+^/Mg^2+^/Zn^2+^) on indirect photodegradation with ·OH. The calculation results show that the direct photodegradation of FLO involves three reaction types (C–C/C–N/C–S bond cleavage). The relatively high *E*_a_ value (94.8 kcal/mol) indicates that C17–C18 cleavage cannot easily occur during the direct photodegradation of FLO. The lower *E*_a_ value (2.0 kcal/mol) indicates that C5–S7 cleavage is the main pathway of the direct photodegradation process. The indirect photodegradation of FLO by free radicals is an addition reaction. The Δ*H* (−41.3 kcal/mol) for adding ·OH at the C5 site is higher than that for other sites on the benzene ring, indicating that the products such as 2,2-dichloro-N-(3-fluoro-1-hydroxy-1-(4-hydroxyphenyl) propan-2-yl) acetamide are the most stable. The indirect photodegradation of FLO by ·NO_3_ and ·SO_4_^−^ is endothermic. The NO_3_-addition reaction is the most likely to occur at the C1 site, while the ·SO_4_-addition reaction is the least likely to occur at the C1 site. The metal ion Ca^2+^ can promote the indirect photodegradation with ·OH, and the metal ion Mg^2+^/ Zn^2+^ has a dual effect on the indirect photodegradation with ·OH. Mg^2+^ can not only promote the ·OH addition reaction at C3 and C4 sites but also inhibit the ·OH addition reaction at C5 and C6 sites. Zn^2+^ can not only promote the ·OH addition reaction at C3, C4, and C6 sites but also inhibit the ·OH addition reaction at C1 site. In addition, in the presence of metal ion Mg^2+^, OH-addition is the most likely to occur at the C4 site, but it is the least likely to occur at the C3 site. These results indicate that the quantum chemical calculation method can be used to study the mechanism of the water-based photochemical transformation of antibacterial drugs and to guide their safe use and risk avoidance.

## Data Availability

Data will be made available on request. The dataset used and/or analyzed in this study is available from the corresponding author on reasonable request.

## Supplementary Materials

The following supporting information can be downloaded at: https://www.mdpi.com/article/10.3390/toxics12020127/s1, Figure S1: Direct photodegradation pathways of FLO, along with computed activation energies (Ea, kcal/mol) and enthalpy change (ΔH, kcal/mol). The numbers in parentheses are NBO charge and electron spin density (NBO charge, electron spin density); Figure S2: Optimized geometries of reactants, transition states, and products in direct photolysis pathways (Path 1, Path 2, Path 3, and Path 4) of FLO, along with selected bond lengths (Å); Figure S3: Optimized geometries of reactants, transition states, and products in indirect photolysis pathways (Path C1, Path C2, Path C3, and Path C4) of FLO with ·OH, along with selected bond lengths (Å); Figure S4: Optimized geometries of reactants, transition states, and products in indirect photolysis pathways (Path C1N, Path C4N, and Path C6N) of FLO with ·NO3, along with selected bond lengths (Å); Figure S5: Optimized geometries of reactants, transition states, and products in indirect photolysis pathways (Path C1S, Path C3S, Path C4S, and Path C6S) of FLO with ·SO4-, along with selected bond lengths (Å); Figure S5: Optimized geometries of complexes FLO–Ca2+/FLO–Mg2+/FLO–Zn2+, along with selected bond lengths (Å); Figure S7: Four optimized geometries (OPT1, OPT2, OPT3, and OPT4) of complex FLO–Ca2+ along with selected bond length (Å). The energies of geometries are relative to that of the most stable geometry FLO–Ca2+–OPT3; Figure S8: Optimized geometries of reactants, transition states, and products in indirect photolysis pathways (Path C3Ca and Path C4Ca) of complex FLO–Ca2+ with ·OH, along with selected bond lengths (Å); Figure S9: Five optimized geometries (OPT1, OPT2, OPT3, OPT4, and OPT5) of complex FLO–Mg2+ along with selected bond length (Å). The energies of geometries are relative to that of the most stable geometry FLO–Mg2+–OPT4; Figure S10: Optimized geometries of reactants, transition states, and products in indirect photolysis pathways (Path C3Mg, Path C4Mg, Path C5Mg, and Path C6Mg) of complex FLO–Mg2+ with ·OH, along with selected bond lengths (Å); Figure S11: Four optimized geometries (OPT1, OPT2, OPT3, and OPT4) of complex FLO–Zn2+ along with selected bond length (Å). The energies of geometries are relative to that of the most stable geometry FLO–Zn2+–OPT1; Figure S12: Optimized geometries of reactants, transition states, and products in indirect photolysis pathways (Path C1Zn, Path C3Zn, Path C4Zn, and Path C6Zn) of complex FLO–Zn2+ with ·OH, along with selected bond lengths (Å).
