# Supplementary material for: Study on the Direct and Indirect Photolysis of Antibacterial Florfenicol in Water Using DFT/TDDFT Method and Comparison of Its Reactivity with Hydroxyl Radical under the Effect of Metal Ions
Source: Toxics. 2024 Feb 3;12(2):127. doi: 10.3390/toxics12020127 (PMC10891592; doi:10.3390/toxics12020127)
Supplement: Supplementary file 1 [file toxics-12-00127-s001.zip › toxics-2837376-supplementary.pdf]

Supplementary Material

Study on the Direct and Indirect Photolysis of Antibacterial Florfenicol in Water Using DFT/TDDFT Method and Comparison of Its Reactivity with Hydroxyl Radical under the Effect of Metal Ions

Yue Kang, Ying Lu and Se Wang *

Collaborative Innovation Center of Atmospheric Environment and Equipment Technology, Jiangsu Key Laboratory of Atmospheric Environment Monitoring and Pollution Control, School of Environmental Science and Engineering, Nanjing University of Information Science and Technology, Nanjing 210044, China

* Correspondence: wangse@nuist.edu.cn; Tel./Fax: +86-25-58731090

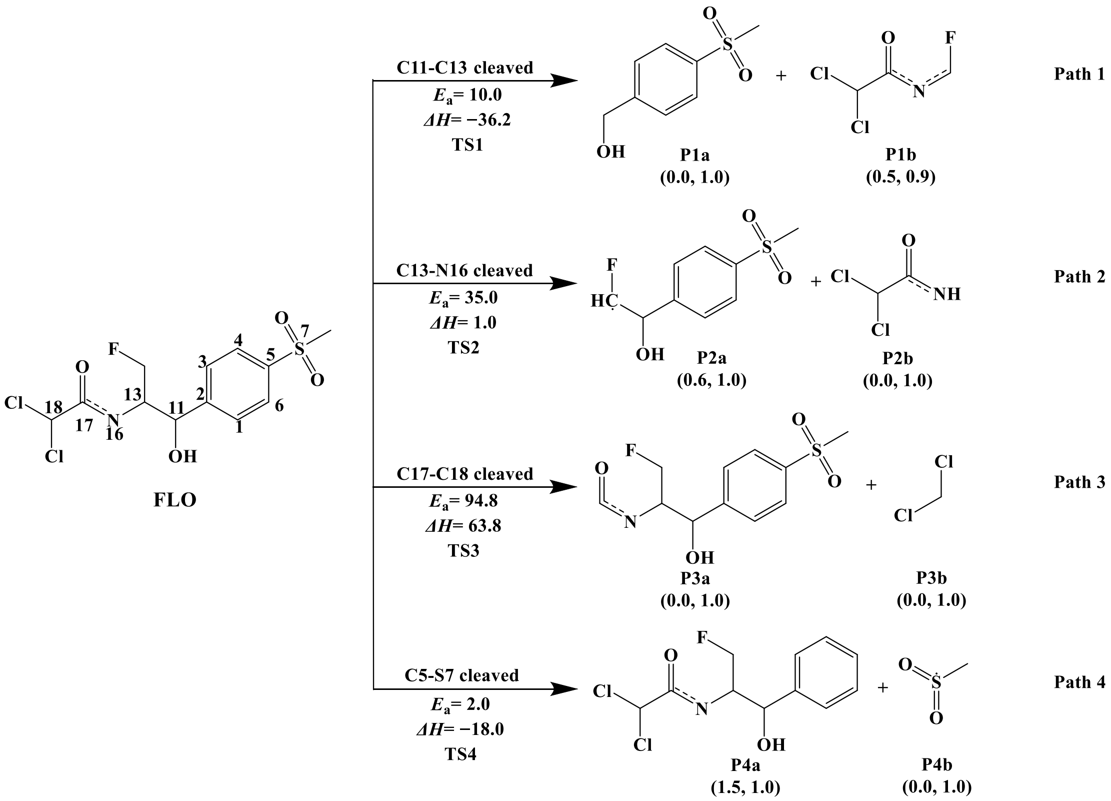

Figure S1. Direct photodegradation pathways of FLO, along with computed activation energies (E_a, kcal/mol) and enthalpy change (ΔH, kcal/mol). The numbers in parentheses are NBO charge and electron spin density (NBO charge, electron spin density).

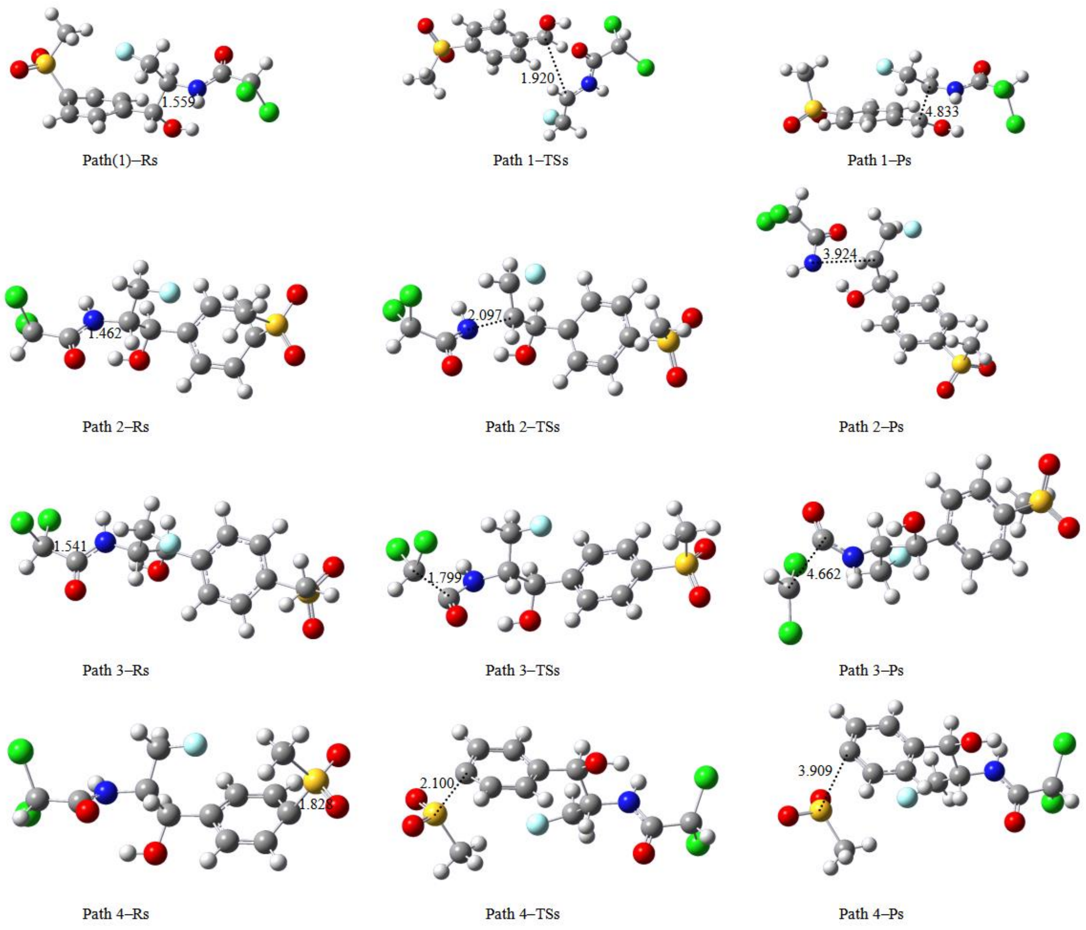

Figure S2. Optimized geometries of reactants, transition states, and products in direct photolysis pathways (Path 1, Path 2, Path 3, and Path 4) of FLO, along with selected bond lengths (Å).

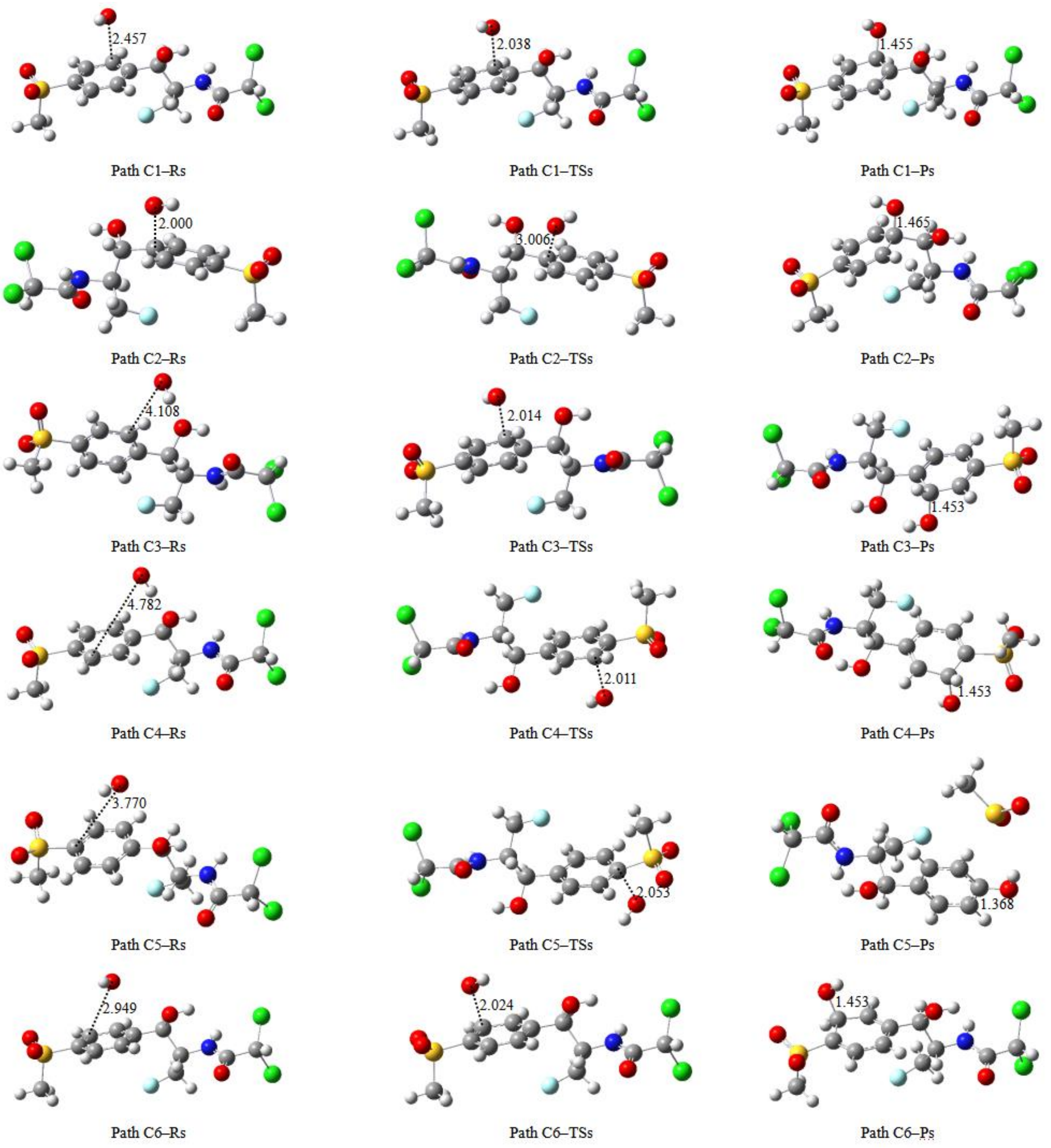

Figure S3. Optimized geometries of reactants, transition states, and products in indirect photolysis pathways (Path C1, Path C2, Path C3, and Path C4) of FLO with ·OH, along with selected bond lengths (Å).

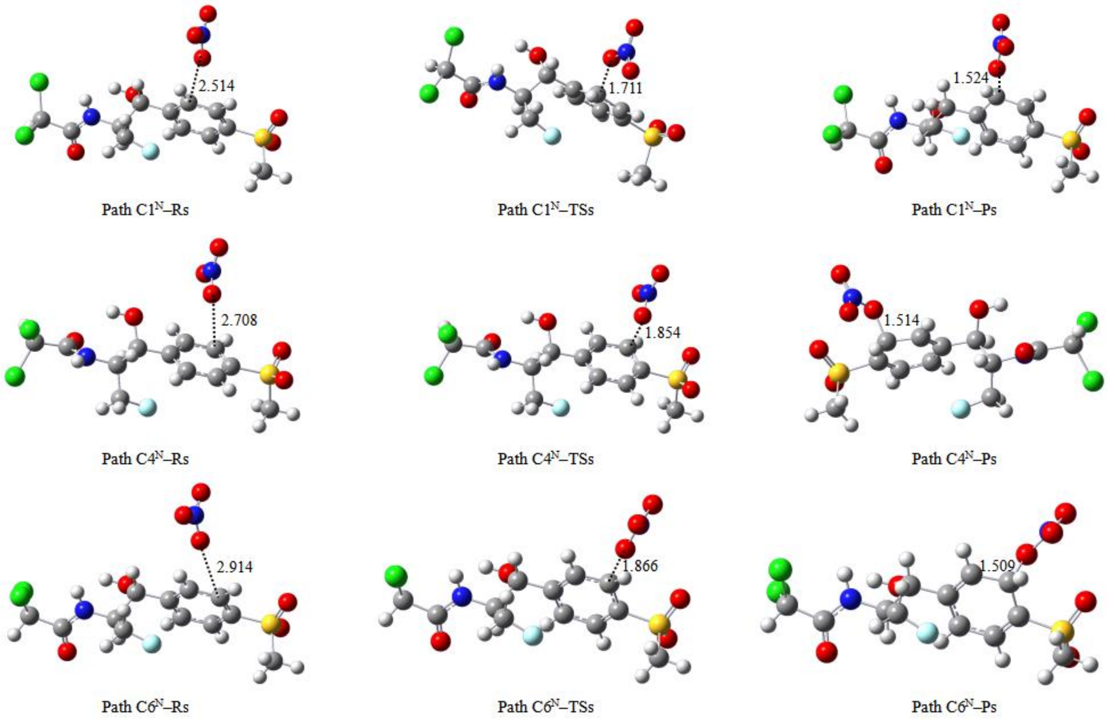

Figure S4. Optimized geometries of reactants, transition states, and products in indirect photolysis pathways (Path $C1^N$, Path $C4^N$, and Path $C6^N$) of FLO with $\cdot NO_3$, along with selected bond lengths (Å).

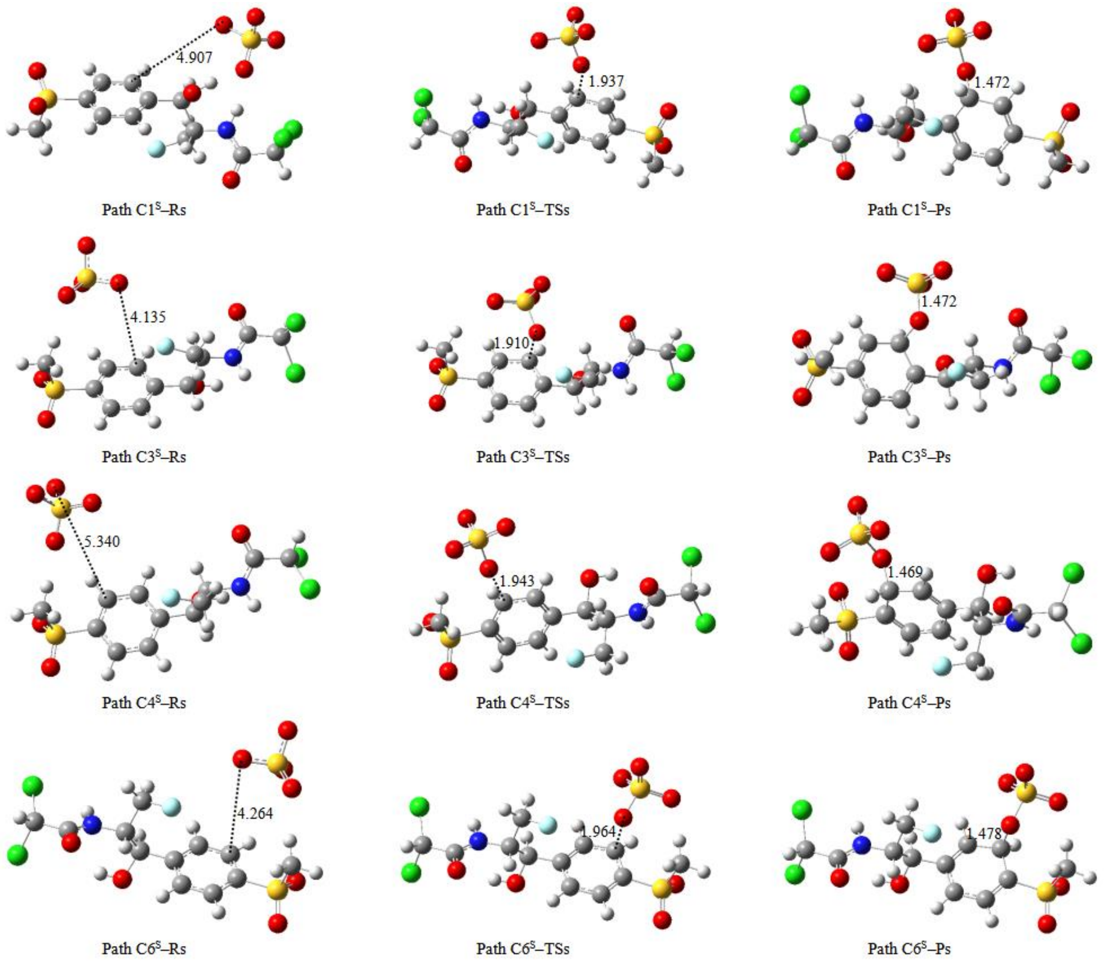

Figure S5. Optimized geometries of reactants, transition states, and products in indirect photolysis pathways (Path C1^S, Path C3^S, Path C4^S, and Path C6^S) of FLO with $\cdot SO_4^-$, along with selected bond lengths (Å).

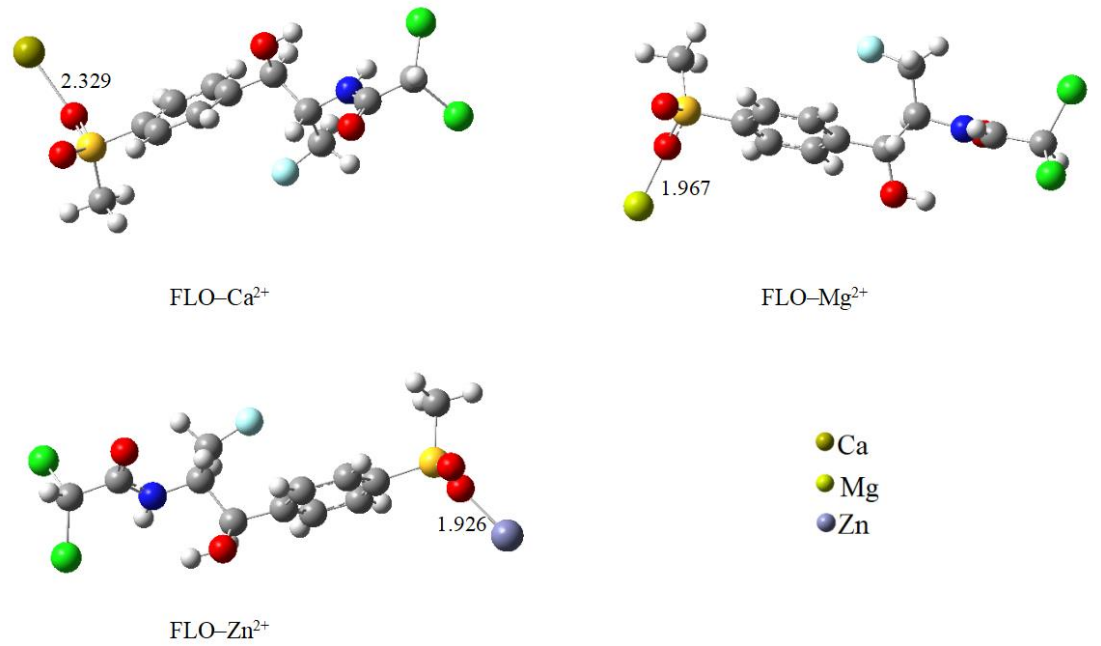

Figure S6. Optimized geometries of complexes FLO–Ca^{2+}/FLO–Mg^{2+}/FLO–Zn^{2+}, along with selected bond lengths (Å).

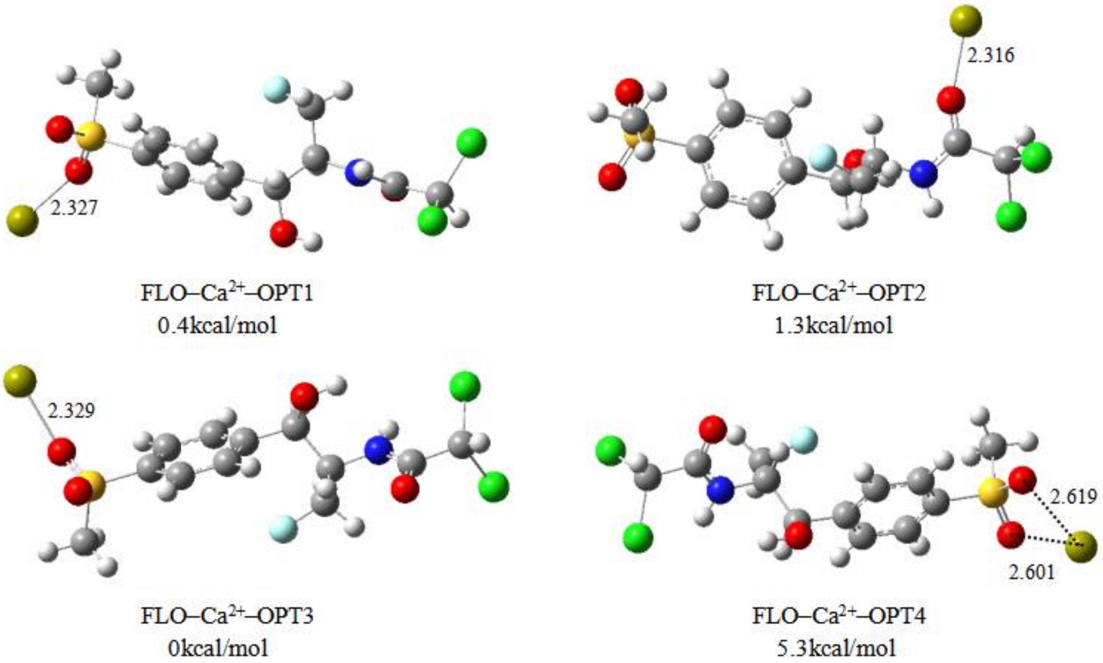

Figure S7. Four optimized geometries (OPT1, OPT2, OPT3, and OPT4) of complex FLO–Ca^{2+} along with selected bond length (Å). The energies of geometries are relative to that of the most stable geometry FLO–Ca^{2+}–OPT3.

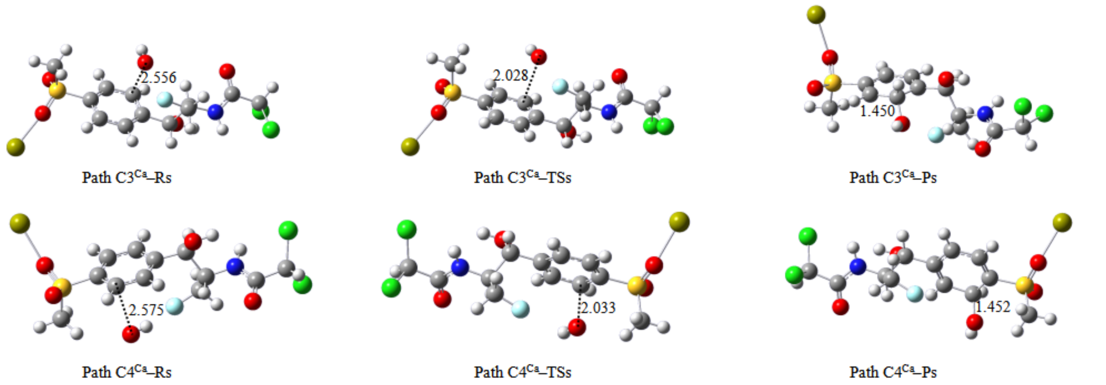

Figure S8. Optimized geometries of reactants, transition states, and products in indirect photolysis pathways (Path $C3^{Ca}$ and Path $C4^{Ca}$) of complex FLO–Ca^{2+} with ·OH, along with selected bond lengths (Å).

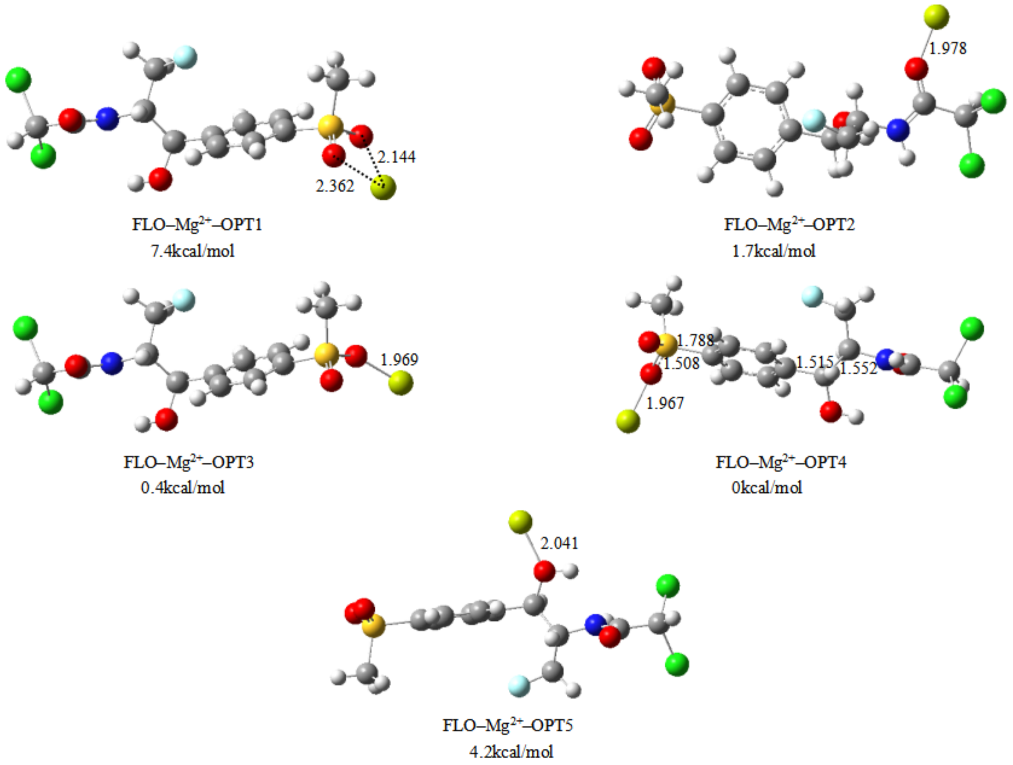

Figure S9. Five optimized geometries (OPT1, OPT2, OPT3, OPT4, and OPT5) of complex FLO–Mg^{2+} along with selected bond length (Å). The energies of geometries are relative to that of the most stable geometry FLO–Mg^{2+}–OPT4.

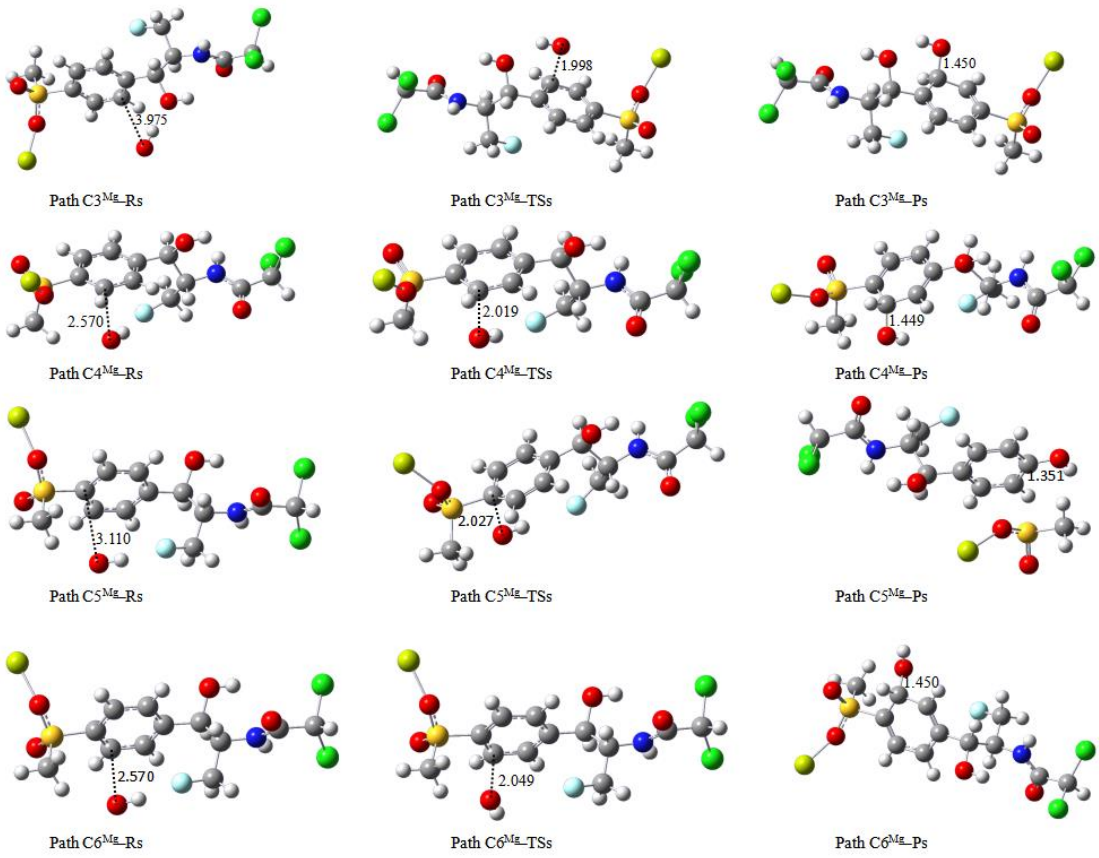

Figure S10. Optimized geometries of reactants, transition states, and products in indirect photolysis pathways (Path C3^{Mg}, Path C4^{Mg}, Path C5^{Mg}, and Path C6^{Mg}) of complex FLO–Mg^{2+} with ·OH, along with selected bond lengths (Å).

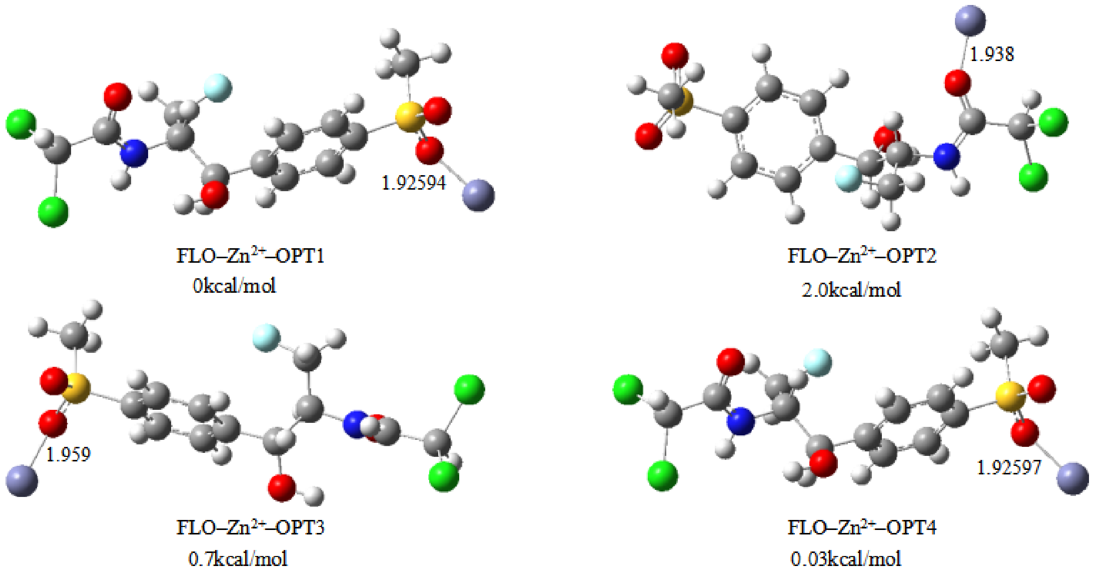

Figure S11. Four optimized geometries (OPT1, OPT2, OPT3, and OPT4) of complex FLO–Zn^{2+} along with selected bond length (Å). The energies of geometries are relative to that of the most stable geometry FLO–Zn^{2+}–OPT1.

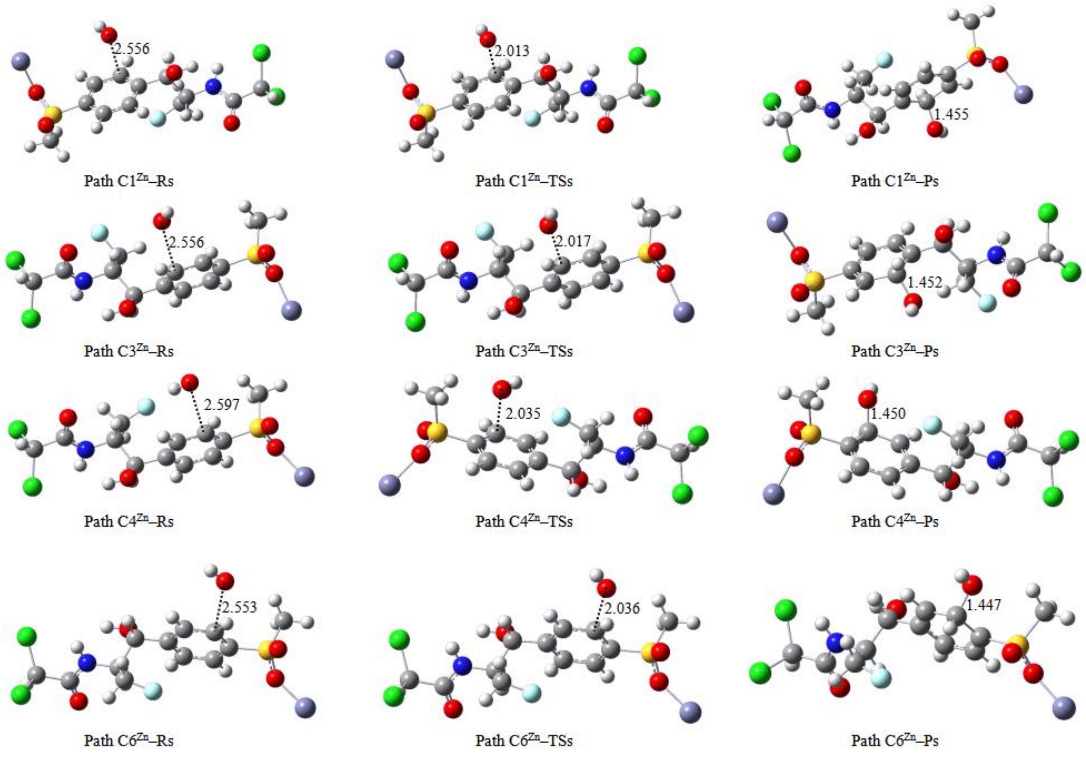

Figure S12. Optimized geometries of reactants, transition states, and products in indirect photolysis pathways (Path $C1^{Zn}$, Path $C3^{Zn}$, Path $C4^{Zn}$, and Path $C6^{Zn}$) of complex FLO–Zn^{2+} with ·OH, along with selected bond lengths (Å).
